# Adapting Equity-Focused Approaches to Address Workflow Challenges During COVID-19: Lessons for the Health Care Workforce

**DOI:** 10.1089/heq.2021.0125

**Published:** 2022-10-12

**Authors:** Brittany M. Chambers

**Affiliations:** Department of Health Equity and Special Initiatives, Center to Advance Palliative Care, Icahn School of Medicine at Mount Sinai, New York, New York, USA.

**Keywords:** health equity, COVID-19, workflow challenges, public health practice, palliative care

## Abstract

The COVID-19 pandemic has severely impacted certain racial and ethnic groups due to systemic racism and poor governmental emergency responses. Health organizations and leaders worked strategically to pivot their workflows to meet the emerging needs of their patient population. In this perspective, three examples are shared of successful interventions that made workflow improvements to be efficient while ensuring excellent patient care during the pandemic and beyond. Lessons from the initiatives are ones that health professionals can advocate for and easily adopt to ensure that medical mistreatment and disparities for some patient populations do not continue to flourish.

## Introduction

A year and a half after the onset of the COVID-19 pandemic, inequities in the U.S. health care system for certain populations are glaringly apparent and can no longer be ignored. When data became available, the public became increasingly aware that racial and ethnic communities such as Black/African Americans, Hispanics, Latinos, and American Indian and Alaska Natives were infected and died at alarming rates compared with their white counterparts. These populations already bore the consequences of structural racism and the lack of a proper emergency preparedness response further exacerbated the poor health outcomes for these groups. At a national organization that provides clinical training and tools for health professionals who treat patients living with a serious/chronic illness, I had an inside view of how health organizations had to reprioritize their workflow and redirect team members to be responsive to the many unexpected changes of the pandemic.

To successfully address existing health inequities and the infrastructures that allow them to persist, we need every single individual in this country to participate in dismantling our inefficient health care system. There is a difference to be made at every level and change is possible within all of our “spheres of influence.” Health professionals have an opportunity to be among the leaders in regaining the trust of the public—especially communities who were disproportionately impacted by the COVID-19 pandemic. There is a unique opportunity to lean into our essential functions and prior training such as advocating for the implementation of models/interventions that will significantly improve care for historically excluded and mistreated patient populations.

In this perspective, I will share three specific examples of health organizations throughout the country (CO, MI, and NY) that had to pivot to ensure care delivery was appropriate for their sickest patients, despite resource scarcity and limited staff. These organizations successfully adopted interventions/workflow improvements to be efficient—and most importantly improve care. They provide important lessons, we as health care advocates can promote/advocate to improve care (particularly in the context of a pandemic) with both genuine intention and vigilance.

## Discussion

Patients with limited English proficiency are more likely to have a poor understanding of their diagnoses, low health literacy, and a greater risk of being misunderstood by their physicians.^1^ At Stony Brook Medicine—a large academic hospital located in New York, one of their physicians shared that with a large influx of Spanish-speaking patients in the midst of the pandemic, they used a model of in-person and virtual interpreters to address language barriers and help reduce disparities in care. With the redesign of their workflow, they were able to identify patients who needed an interpreter earlier and do system-wide staff education and training, which led to improved patient experience. With the use of interpreters, 30 in-person interpreters for high influx areas and virtual interpretation services via donated tablets and partnership with IT vendors, they were able to address a gap in need.^2^

More than 1000 of Stony Brook's patients used the interpreters during the height of the pandemic. This initiative is important for health professionals to be aware of because it shows the feasibility and replicability for this model to be utilized in similar settings. This approach not only improves patient experience but also helps clinical teams tap into interpreters as an extension of the care team and utilize them as an essential resource during challenging times and on an ongoing basis.

As health equity advocates, which all health professionals should be—this second example acknowledges and works to address inequities for Black patients living with a serious illness. Recent literature shows that Black patients are less likely to be believed when they report pain, three times more likely than white patients to say that their end-of-life preferences were not taken into account by their clinicians, and report being less satisfied with the quality of provider communication (a hallmark element of navigating a serious or chronic illness).^3^

At the Henry Ford Hospital in Detroit, MI, 78% of their patients identify as Black/African American and more than one-third of their patient population are impoverished.^4^ In knowing these statistics, social determinants of health considerations had to be made. As one of the worst affected areas in the country early in the COVID-19 pandemic, clinicians at the Henry Ford Hospital observed that the team (palliative medicine team) that was equipped to have high-quality goals-of-care conversations about the conditions and expertly manage distressing symptoms was called in after the patients were intubated or close to death.

Consequently, this led to inappropriate procedures/use of scarce resources, increased health care costs, and more importantly, increased suffering for Black patients. The team decided to intervene by seeing high-risk patients upon arrival to the emergency department (ED), to increase patient participation in goals-of-care discussions earlier and get their wishes and preferences for care documented before being admitted.^4^ By embedding a trained palliative care specialist in the ED during the pandemic, they were able to successfully support other providers (reduce burnout), increase patient autonomy, allow families time to discuss preferences, and allocate hospital resources to patients who wanted life-sustaining treatments. This example showcases the importance of redirecting appropriate staff for earlier interactions and uses a more proactive approach.

This model can also be used with other types of care professionals and during nonpandemic times. For example, embedding a community health care worker in the ED to work as a trusted liaison to help Black patients and their caregivers navigate the system and advocate with them to match their preferences to services received.^5^ These existing types of models have shown promising results such as reduction in ED visits and unnecessary hospital utilization, better care plans, and an overall improved quality of life.

The last example comes out of the University of Colorado Hospital (UCH). The racial and ethnic breakdown in the UCH-defined community is 62% white and 24% Hispanic.^6^ Despite this breakdown, the majority of the COVID-19-positive patients seen during the pandemic were disproportionately Black and Hispanic, with Hispanics making up 38% of the COVID cases.^7^ The pandemic served as a catalyst and highlighted the need for upstream identification of medical decision makers to ensure that patients identify someone they trust to make decisions for them, if necessary. This example integrated a palliative care physician and social worker, who are trained in navigating challenging conversations into the ED for three weeks. Due to the earned mistrust by the medical system, historically oppressed patient populations with documented trusted decision makers can help increase the likelihood that their care preferences will be met.

The initiative was found to be successful as patients with a documented medical decision maker increased from 9% to 86% during that time frame.^8^ In addition, a survey showed that 80% of patients were proactively identified as needing more upstream symptom management services. This finding is important in addressing existing health disparities and connecting high-risk patients to services that could drastically improve their quality of life. Ninety-eight percent of ED staff survey respondents felt that the embedded model provided better patient care and it was easy to incorporate into their workflow.

Two important themes emerged from this example for health professionals to pay attention to. One, by adding a dedicated team member to the ED, they were able to increase their documentation of a decision maker by 77%, showing a major quality improvement opportunity. Second, ED staff sharing that the initiative was both beneficial for patients and did not disrupt their workflow is an exceptional and promising finding on its viability. It demonstrates that with creativity and willingness to pilot evidenced-based models, patient experience can be drastically improved for Black and Hispanic patients. We can begin to make significant strides to ensure that these patient populations feel heard and respected by the medical system and their care preferences are prioritized.

## Conclusion

Since health professionals work in various capacities, in a plethora of settings they are uniquely positioned to respond to pandemics similar to COVID-19. For instance, they can be influential in how health systems reprioritize workflows and redirect their staff to respond to national emergencies/crises. They can also help increase the access and availability of health care services to the community, indirectly helping to address the overall health disparities. Consequently, this proactive approach can ensure that we do not see the same disproportionate suffering of certain groups, the way we did for COVID-19, in the future.

Those in leadership positions who can influence the infrastructure should be aware of nontraditional models such as the ones shared in this perspective to address staffing issues or gaps (e.g., virtual interpreters, palliative care clinicians). Being aware of the impact of these sorts of interventions with positive outcomes, health professionals can advocate for financial support to sustain these services long term or to scale them up sufficiently in times of emergencies.

Finally, health practitioners can serve as a bridge to community stakeholders that work with historically excluded and underserved communities. This should remain a priority and an ongoing part of emergency preparedness/response planning. Stakeholders should include community health centers, payers/insurance providers, policy makers (state and federal), community-based organizations, and faith-based institutions. This ongoing coordination would be valuable in planning an efficient emergency response, identifying gaps, developing culturally responsive messaging materials, and a thoughtful dissemination strategy. Health care workers play key roles in the current pandemic and will be instrumental in utilizing strategic tactics to prevent and/or minimize future health crises and the effect on disproportionately impacted communities.

## Abbreviations Used

ED: emergency department
UCH: University of Colorado Hospital
